# Supplementary material for: Apremilast improves cardiomyocyte cohesion and arrhythmia in different models for arrhythmogenic cardiomyopathy
Source: Stem Cell Res Ther. 2025 Nov 4;16:609. doi: 10.1186/s13287-025-04755-y (PMC12584335; doi:10.1186/s13287-025-04755-y)
Supplement: Supplementary file 1 — Supplementary Material 1. [file 13287_2025_4755_MOESM1_ESM.pptx]

## Slide 1
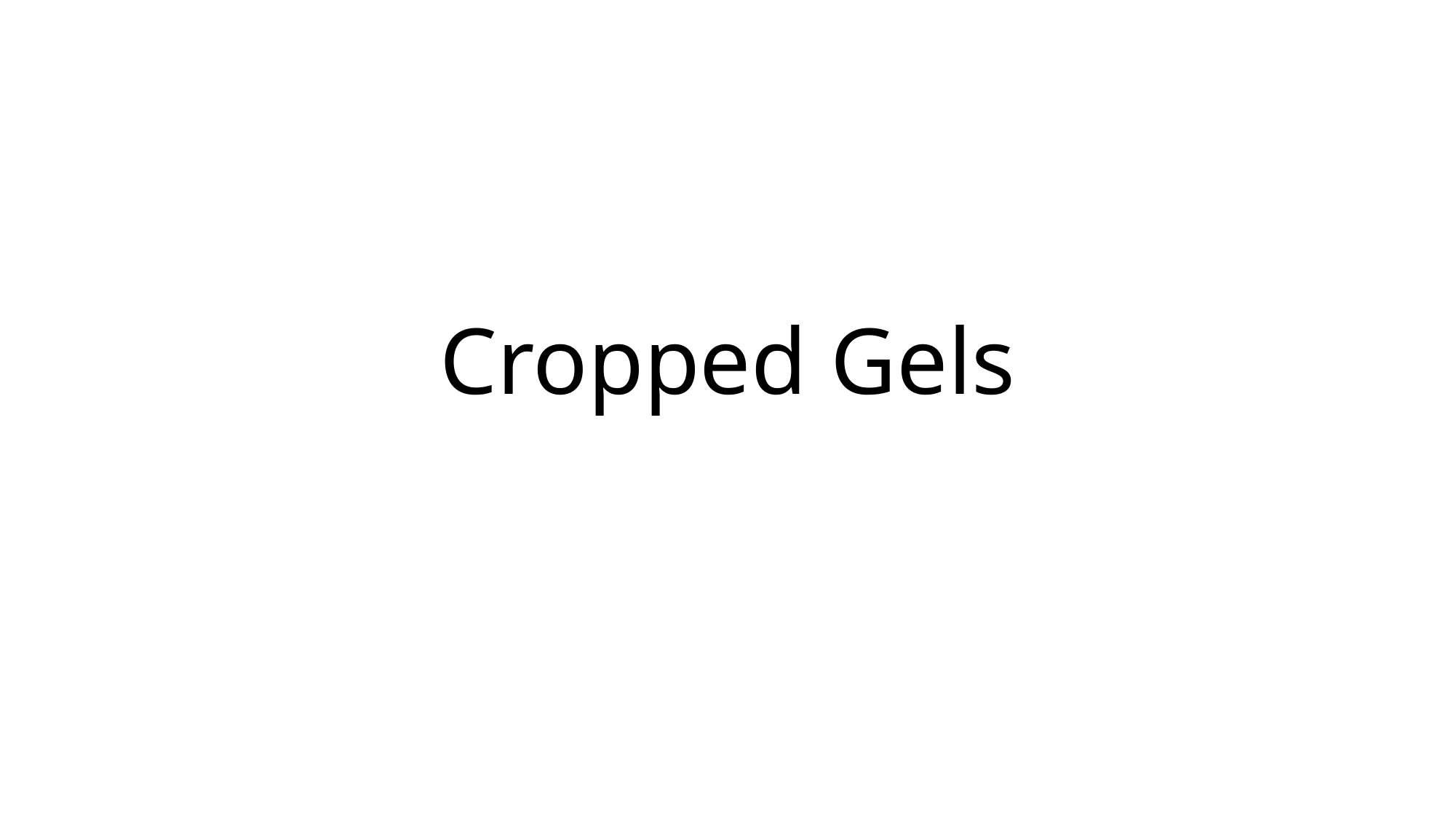

# Cropped Gels

## Slide 2
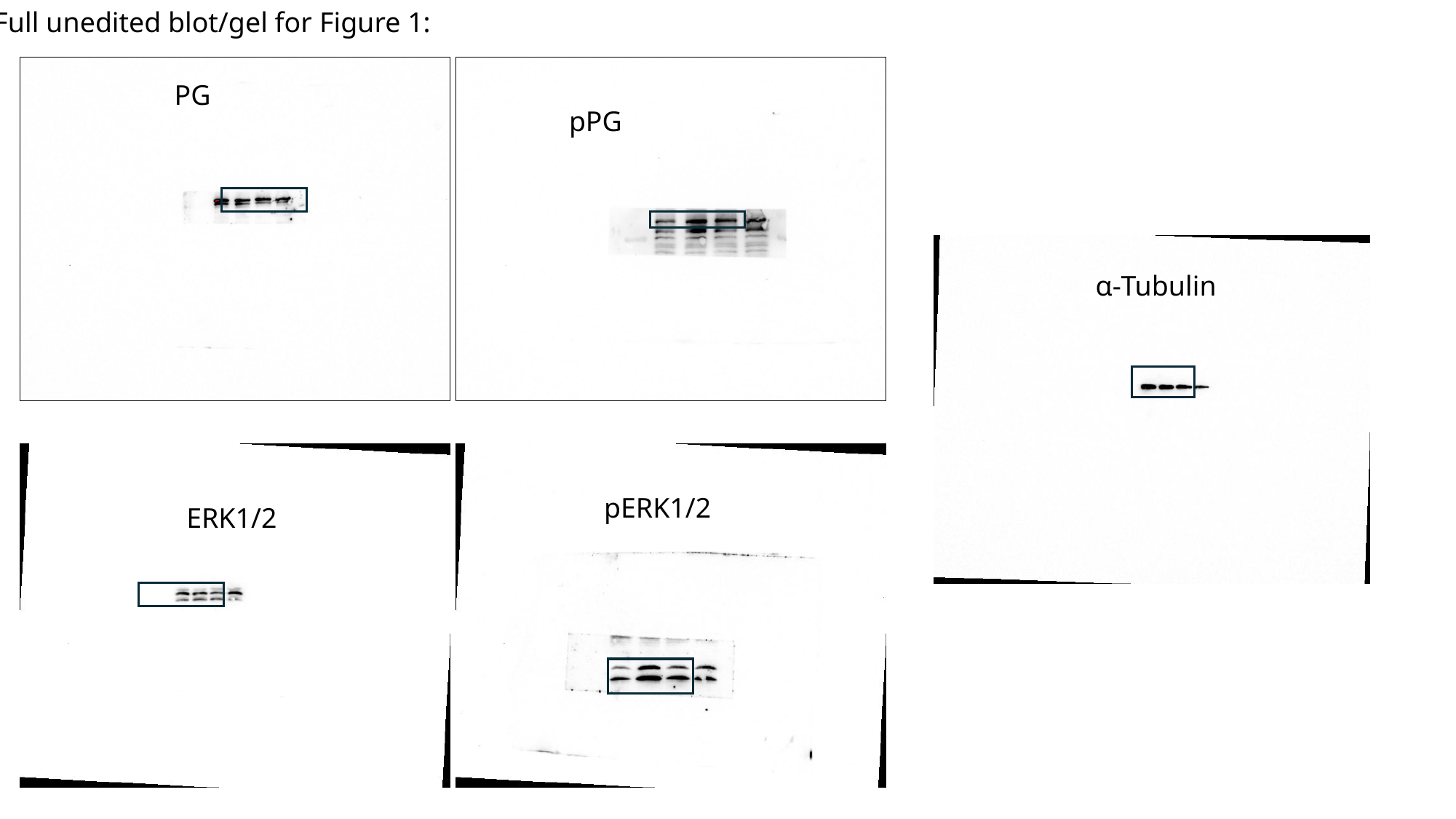

Full unedited blot/gel for Figure 1:
PG
pPG
α-Tubulin
pERK1/2
ERK1/2

## Slide 3
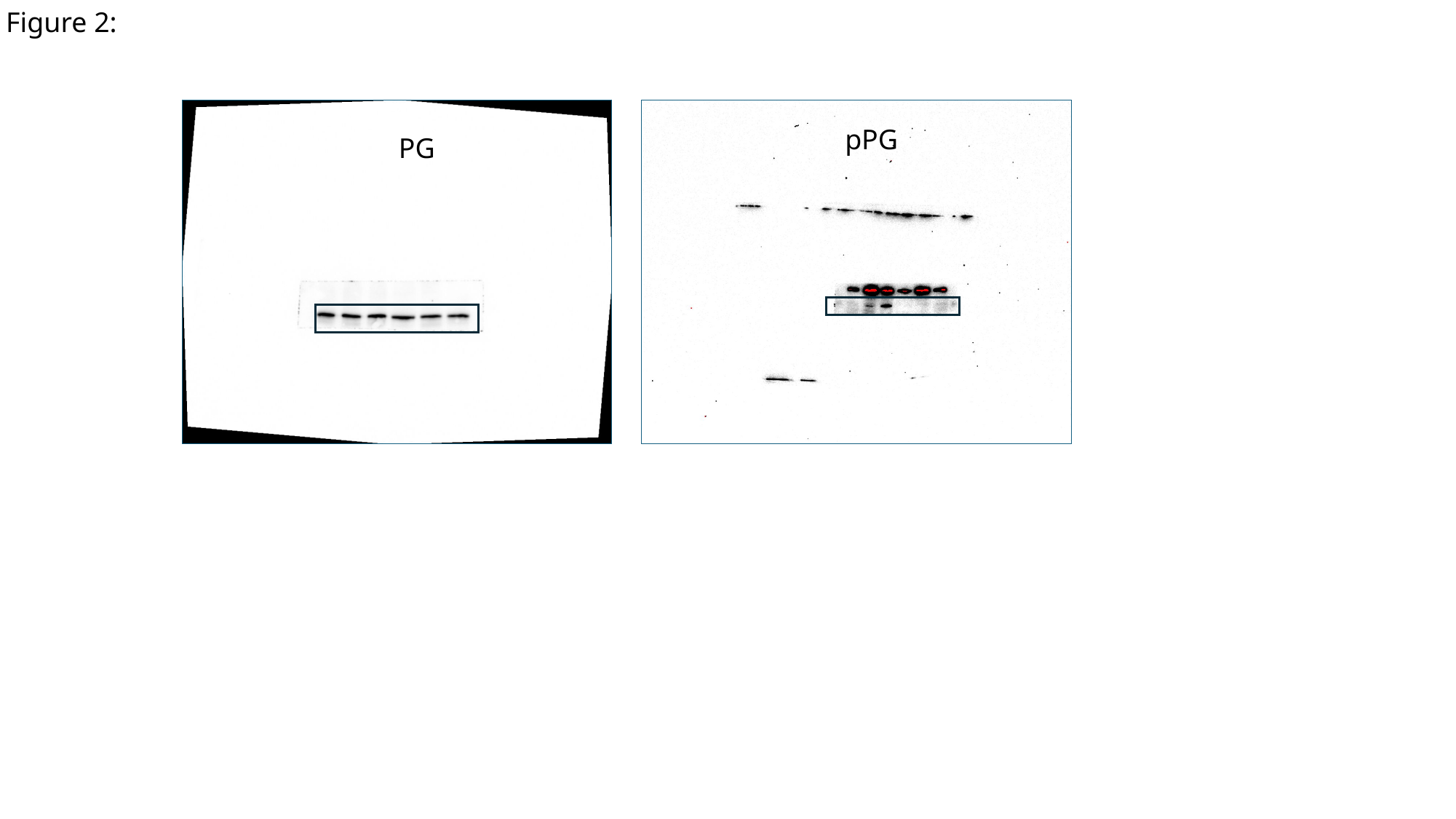

Figure 2:
pPG
PG

## Slide 4
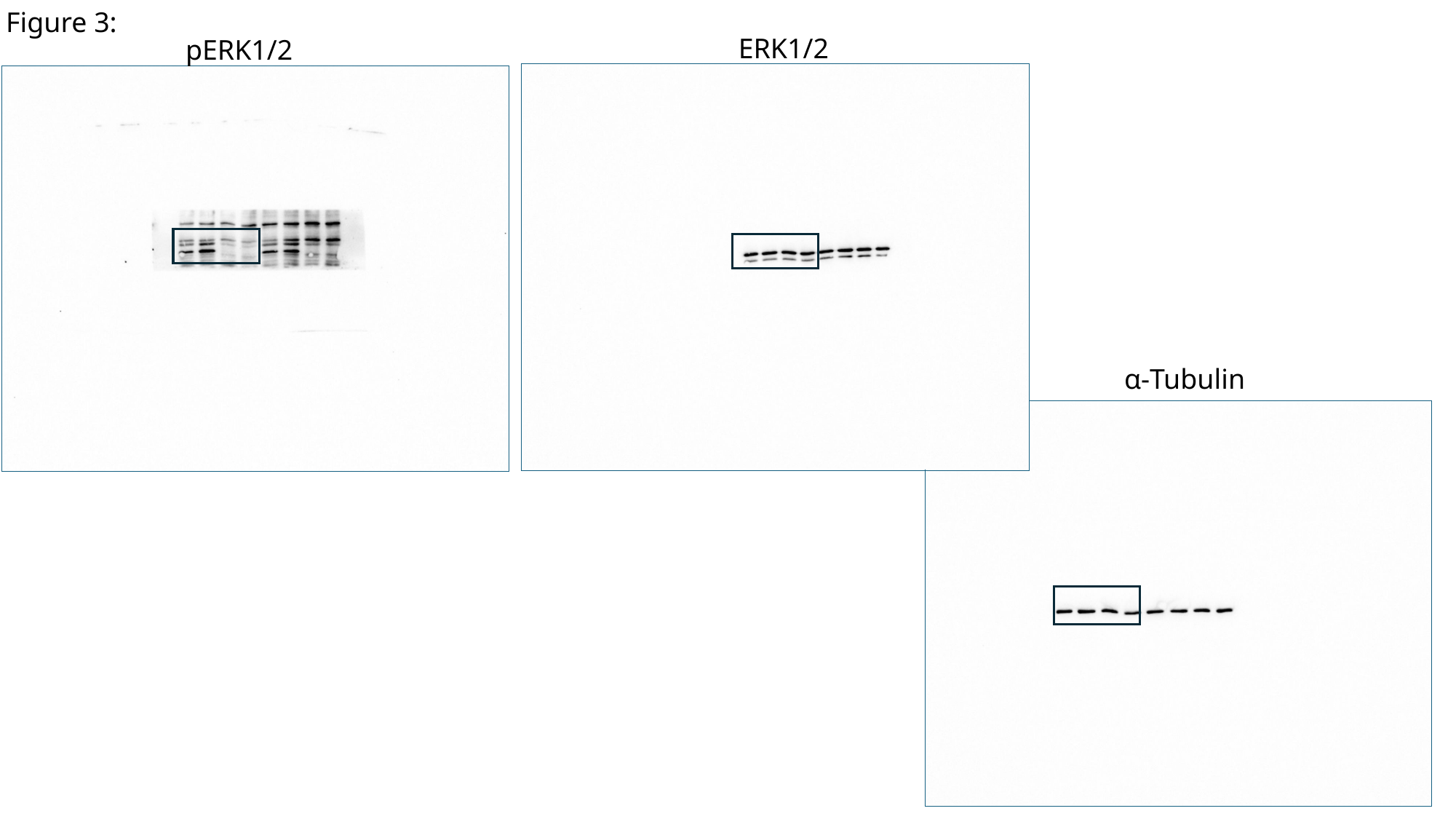

Figure 3:
ERK1/2
pERK1/2
α-Tubulin

## Slide 5
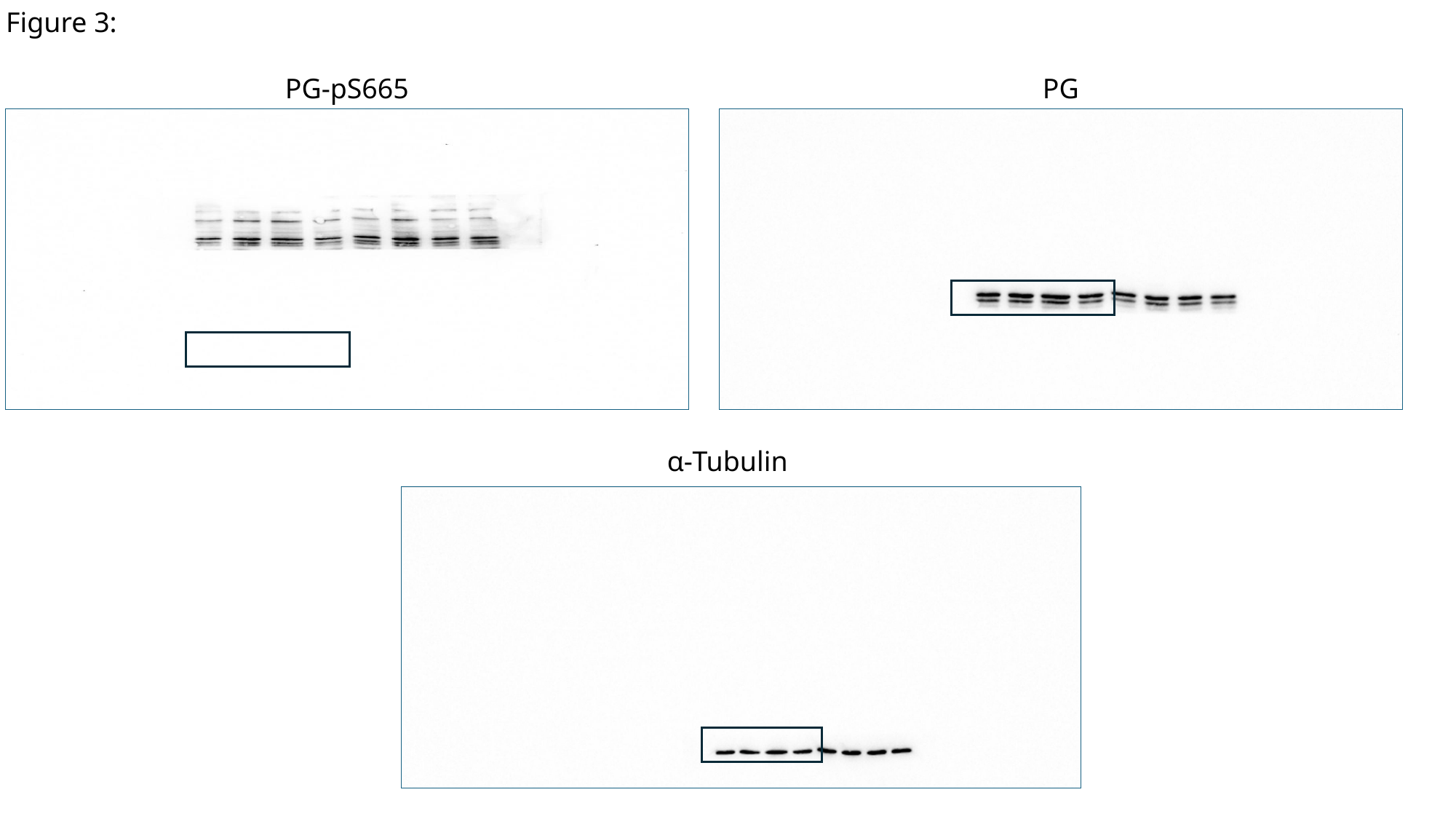

Figure 3:
PG-pS665
PG
α-Tubulin

## Slide 6
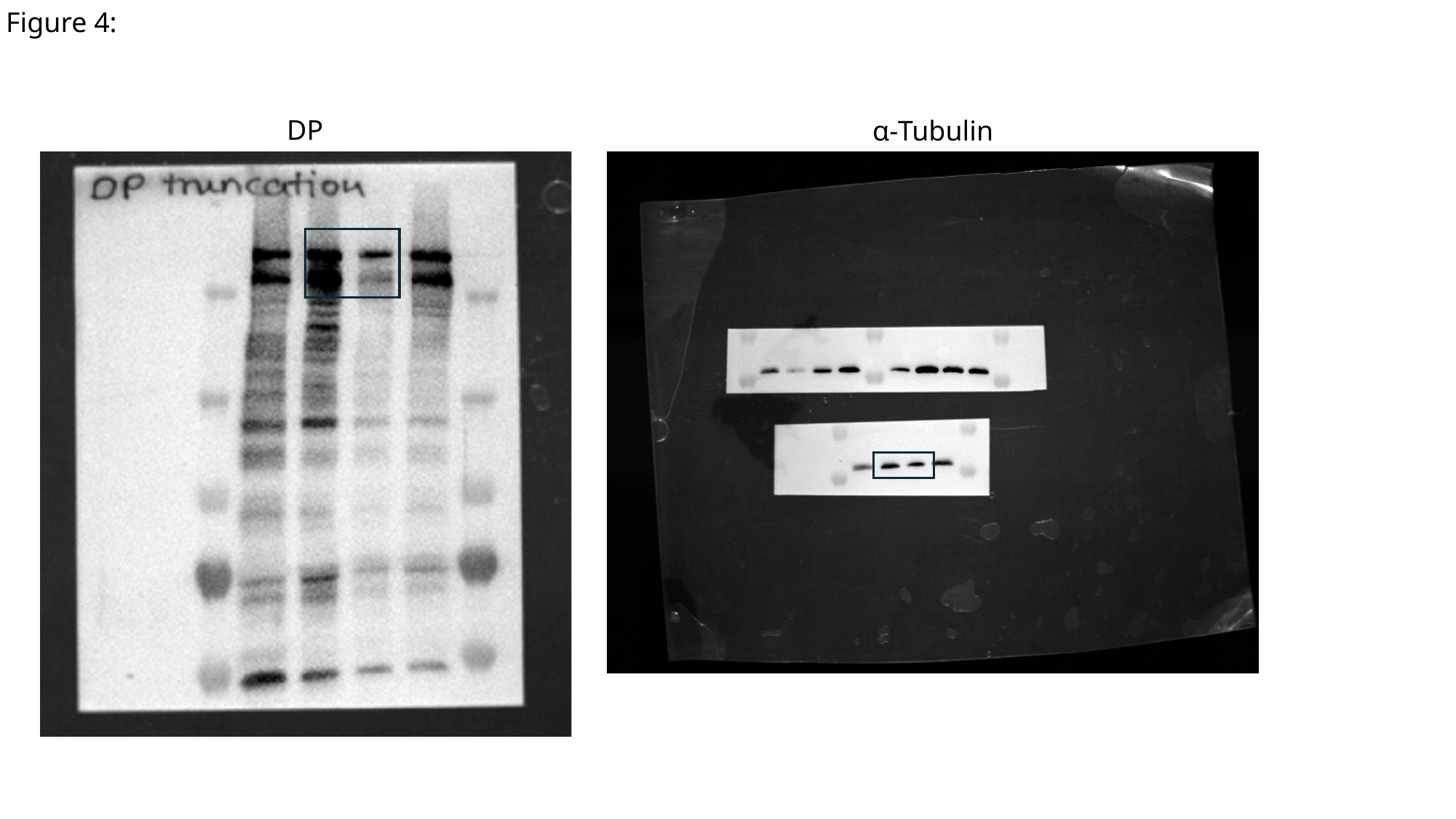

Figure 4:
DP
α-Tubulin

## Slide 7
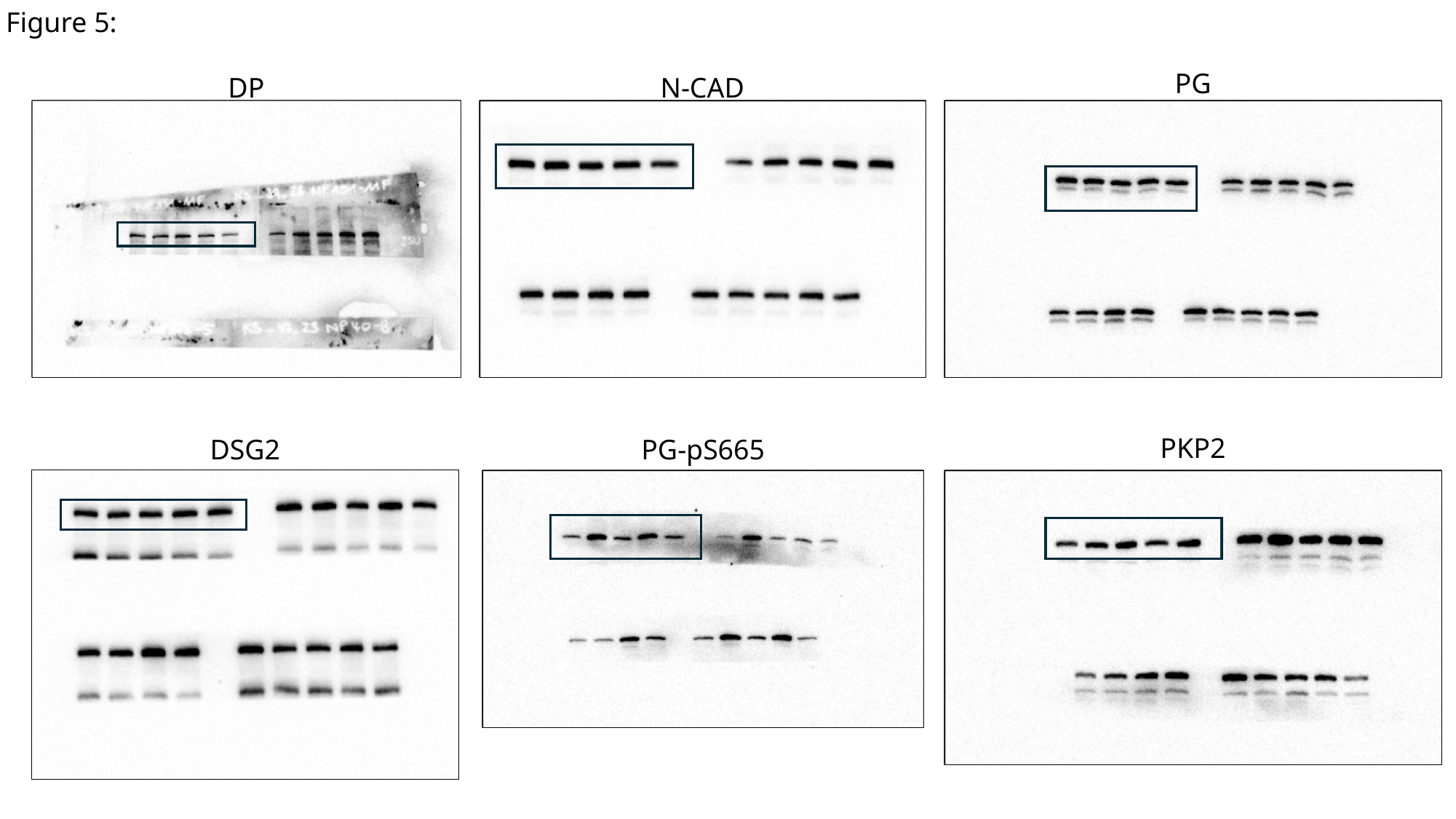

Figure 5:
PG
DP
N-CAD
PKP2
DSG2
PG-pS665

## Slide 8
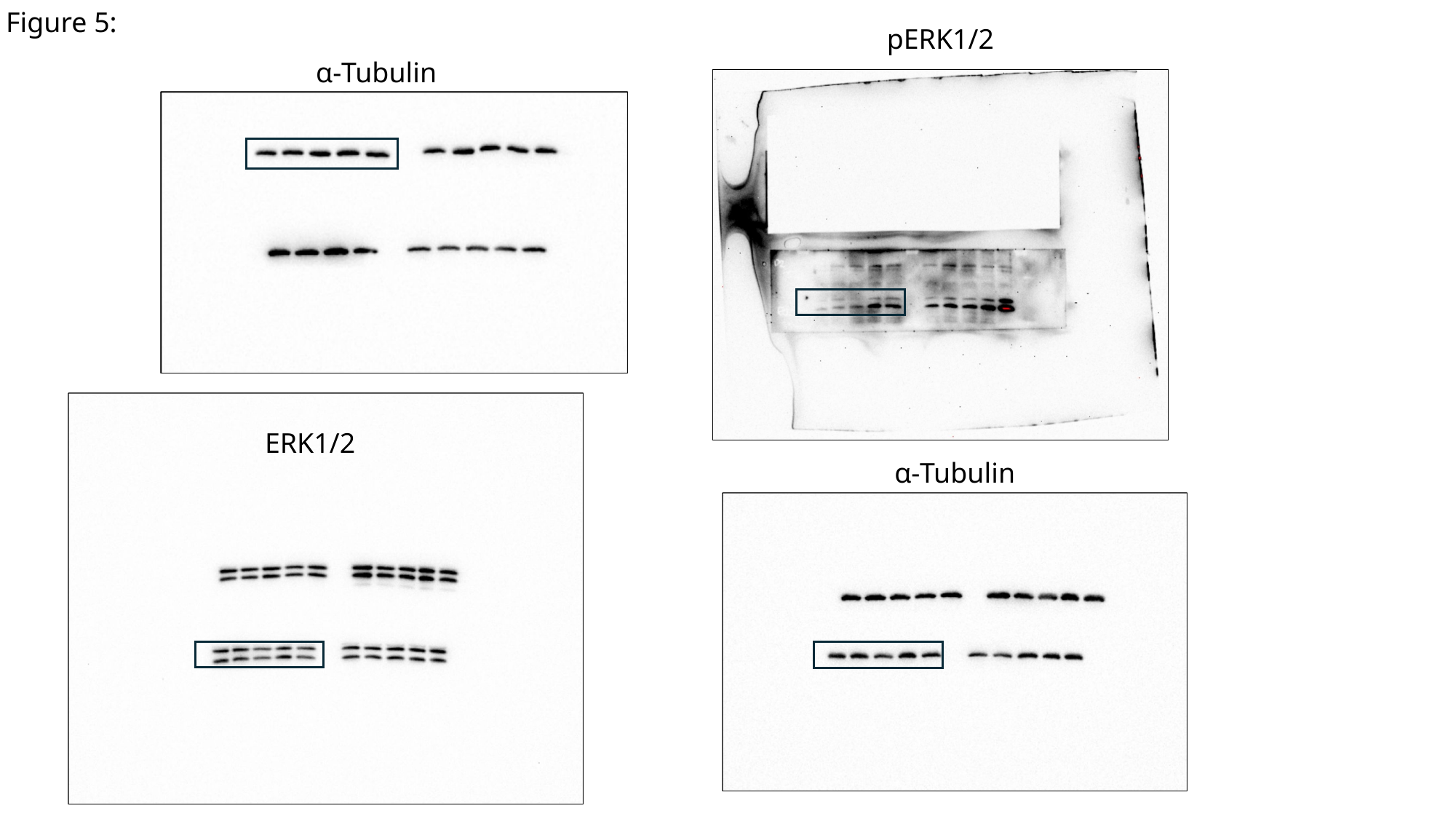

Figure 5:
pERK1/2
α-Tubulin
ERK1/2
α-Tubulin
